# Generating Recombinant Antibodies to Membrane Proteins through Phage Display

**DOI:** 10.3390/antib5020011

**Published:** 2016-05-02

**Authors:** Renhua Huang, Margaret M. Kiss, Melissa Batonick, Michael P. Weiner, Brian K. Kay

**Affiliations:** 1 Department of Biological Sciences, University of Illinois at Chicago, Chicago, IL 60607-7060, USA; rhuang@meso-scale.com; 2 AxioMx Inc., a subsidiary of Abcam Plc, Branford, CT 06405, USA; margaret.kiss@abcam.com (M.M.K.); melissa.batonick@abcam.com (M.B.); michael.weiner@abcam.com (M.P.W.)

**Keywords:** affinity selection, fragments of antigen binding (Fabs), G-protein coupled receptors (GPCRs), membrane proteins, nanodiscs, phage-display, recombinant antibodies, single-domain antibodies, transfection, virus-like particles (VLPs)

## Abstract

One of the most important classes of proteins in terms of drug targets is cell surface membrane proteins, and yet it is a challenging set of proteins for generating high-quality affinity reagents. In this review, we focus on the use of phage libraries, which display antibody fragments, for generating recombinant antibodies to membrane proteins. Such affinity reagents generally have high specificity and affinity for their targets. They have been used for cell staining, for promoting protein crystallization to solve three-dimensional structures, for diagnostics, and for treating diseases as therapeutics. We cover publications on this topic from the past 10 years, with a focus on the various formats of membrane proteins for affinity selection and the diverse affinity selection strategies used. Lastly, we discuss the challenges faced in this field and provide possible directions for future efforts.

## 1. Introduction

Recombinant affinity reagents offer many practical advantages compared to polyclonal or monoclonal antibodies [1]. First, as sequenced entities, they are renewable and easily shared as digital files (*i.e.*, DNA sequences). Second, they are amenable to genetic engineering for epitope tagging and affinity maturation [2]. Third, they can be produced in sufficient quantities for thorough validation. Fourth, they can be generated through phage-display using high-throughput methods [3,4,5]. Across the world, there have been numerous efforts in improving technologies for proteome-scale production of recombinant affinity reagents [3,4,5,6,7,8,9,10], based on the expectation that, in the future, affinity reagents will be principally recombinant in nature. 

The most common form of recombinant affinity reagents is the antibody fragment. This consists of single-chain Fragments of variable regions (scFvs), which are single polypeptides composed of two variable domains of the light and heavy chains of immunoglobulin G (IgG) molecule [11,12], or Fragments of antigen binding (Fabs), which consist of the entire light chain, and the N-terminal variable domain and first constant region of the heavy chain [13,14,15]. Another popular antibody fragment for phage-display is the single-domain antibody, also called the “nanobody”, which corresponds to the variable domain of the camelid heavy-chain antibody [16,17,18]. Other types of protein scaffolds, also currently in use as recombinant affinity reagents, include anticalins [19], the affibodies [20], designed ankyrin repeat proteins (DARPins) [21], and fibronectin type III (FN3) monobodies [22].

**Figure 1:**
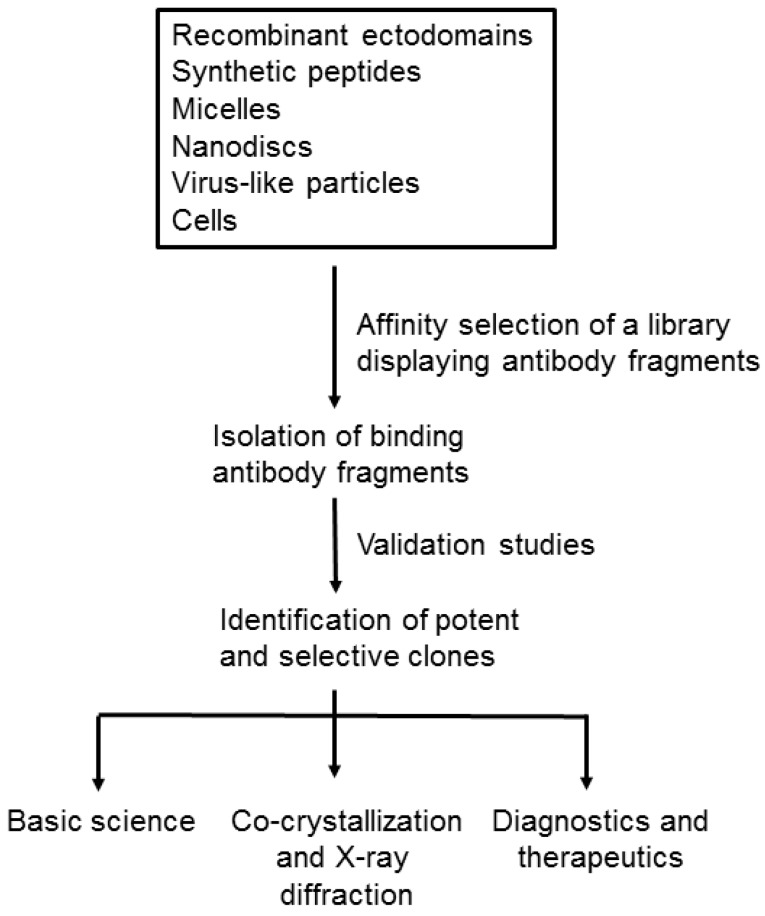
Workflow for generating recombinant antibodies to membrane proteins. Purified membrane proteins, presented in different formats, are mixed with a library of virions displaying antibody fragments. The antibody binders are selected by affinity selection and the encoded antibody fragments are characterized biochemically. The most potent and selective binders are chosen for various applications, such as basic science, crystallography, diagnostics and therapeutics.

Over the years, phage-display technology has matured and techniques for constructing large libraries have improved. Consequently, it is straightforward to generate a recombinant affinity reagent to virtually any soluble and well-folded proteins. One class of targets that has been very challenging for phage-display experiments is the membrane protein. Generally, these proteins are difficult to overexpress in large amounts, and their stability normally requires the presence of artificial detergents or membranes. These challenges have led to a number of innovative methods for formatting membrane proteins as targets for phage-display experiments and affinity selection (Figure 1): they include recombinant proteins and synthetic peptides, detergent micelles, “nanodiscs", virus-like particles (VLPs), and intact cells. Work with each of these formats is described in more detail below.

## 2. Formats of Membrane Proteins for Affinity Selection

### 2.1. Recombinant Proteins and Synthetic Peptides

**Figure 2:**
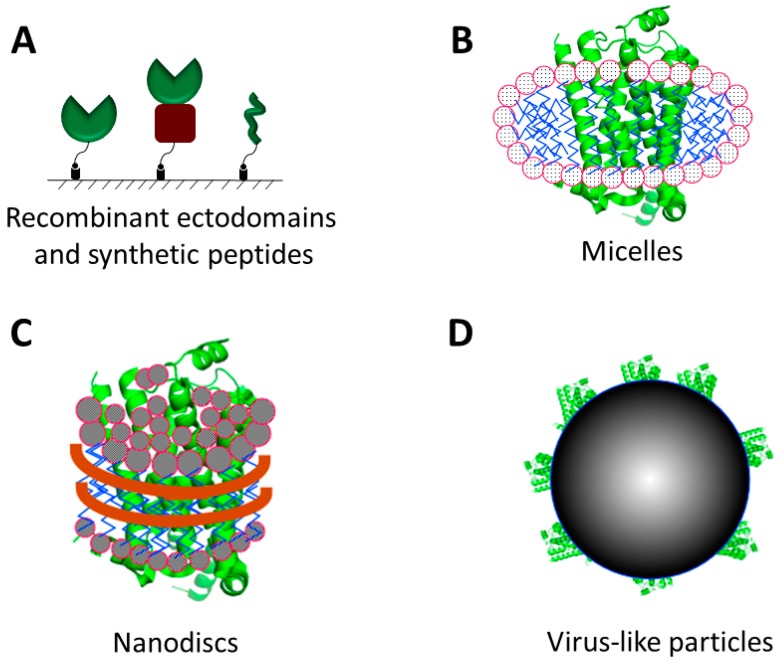
Different formats for presenting membrane proteins for affinity selection experiments. (**A**) The extracellular domains (green) of membrane proteins can be expressed alone or fused to another protein, such as Fc region of immunoglobulin G (IgG) (dark red). Portions of membrane proteins can also be chemically synthesized as peptides (green). These reagents can then be biotinylated and immobilized on streptavidin-coated surfaces (*i.e.*, streptavidin-coated magnetic beads) for affinity selection. (**B**) Membrane proteins can be solubilized with detergents, which form micelles around the membrane proteins (e.g., G-protein coupled receptors shown here). (**C**) Apolipoproteins wrap around phospholipid molecules to form lipid-bilayer nanodiscs, in which membrane proteins insert. (**D**) The membrane proteins of interest can be displayed on the surface of virus-like particles (VLPs), which are sturdier than mammalian cells and permit harsh washing conditions during affinity selection.

Membrane proteins often express poorly in heterologous cells [23]. One approach to circumvent this bottleneck is to express their extracellular domains (*i.e.*, ectodomains) as soluble and secreted fusion proteins (Figure 2A) in mammalian [24,25,26,27], bacterial [28,29,30,31], yeast [32,33] and insect cells [27,34]. For example, as long as the fusion partner does not interfere with proper folding of the ectodomains, they can be expressed fused to the Fc region of IgG or an affinity tag, such as the His_6_-tag [35] and FLAG tag [36]. (Multi-pass membrane proteins, which lack a single ectodomain, cannot be overexpressed in this manner.) It should be noted that ectodomains overexpressed in bacterial systems will lack the post-translational modifications commonly found on mammalian membrane proteins.

Multi-pass membrane proteins, such as G-protein coupled receptors (GPCRs), are even more challenging to overexpress due to their high conformational complexity and low stability in heterologous cells. Multiple methods have been developed for improving the expression and stability of such membrane proteins [37,38,39]. In the first method, stable hydrophilic protein domains are fused to the intracellular loops of GPCRs to improve their expression and promote crystallization [37,40]. T4 Lysozyme (T4L) is the first and most widely used domain that has been used to engineer several GPCRs for crystallization [37,40,41,42,43]. Recently thermostabilized apocytochrome b_562_RIL (BRIL) has been used successfully to engineer well-expressed GPCR for crystallization [44,45]. In the second method, GPCR mutants were generated by alanine scanning [38,46] or leucine scanning [47], and were expressed in *Escherichia coli* (*E. coli*) cells. Mutants with higher stability were screened by binding assay with radiolabelled ligands after heat treatment. This is a simple and straightforward method and does not require special equipment for performing the assays. However, generating hundreds of individual alanine or leucine mutants can be time-consuming, thus this approach is not well-suited to high throughput. In the third method [39], GPCR mutants are created by error-prone PCR, and mutants with random mutations are expressed on the inner membrane of *E. coli* cells and screened by fluorescence-activated cell sorting (FACS) [48] with a fluorescent ligand. This method is predisposed to a much higher throughput, and multiple GPCRs with improved expression and stability [39,49,50] have been engineered. GPCR mutants with higher expression and stability engineered by the above three methods are useful for crystallization [51,52], biophysical characterization, and drug screening [53].

In lieu of overexpressed membrane proteins, short peptides (*i.e.*, <15 amino acids) can serve as surrogate targets. They can be ordered from commercial vendors, and readily used in affinity selection experiments for generating recombinant antibodies to membrane proteins [54,55,56]. One drawback of using peptides as targets are that they represent linear, and not conformational, epitopes of the membrane protein and that such epitopes may not be accessible in the native state of the protein. Consequently, regions of membrane proteins that are thought to be extended, such as the N- and C-termini and loop sequences, are generally chosen for peptide design.

For selection of binders from a phage-displayed antibody library, the target protein or peptide typically contains an added biotin moiety, which permits simple capture by a streptavidin-coated magnetic bead. Alternatively, the target carries an epitope or fusion partner [57,58] that serves as a handle for recovery. Direct capture of the membrane protein to a solid support, such as a polystyrene well of microtiter plate, is problematic, as such direct absorption often leads to protein denaturation [59].

### 2.2. Detergent Micelles, Liposomes and Nanodiscs

Due to the hydrophobicity of their transmembrane regions, membrane proteins often misfold, denature, or aggregate when placed in an aqueous solution free of phospholipids. Therefore, for creating a membrane-like environment to preserve the stability and integrity of membrane proteins, membrane proteins are purified in the presence of detergents, excess of which are then dialyzed away for the subsequent studies [60]. Detergents form micelles (Figure 2B) when their concentrations reach or exceed their critical micelle concentration (CMC) [61]. Micelles-bearing membrane proteins have been used successfully to isolate phage-displayed antibodies to the sodium-citrate co-transporter [62,63] and to isolate monobodies to the fluoride ion channel [64]. However, applications of detergent micelles are limited to due to their instability and size heterogeneity, which may cause some solubilized membrane proteins to unfold and aggregate [65]. A second method for solubilizing and stabilizing the membrane protein is to insert them into liposomes [60,66,67], a bilayer structure that more closely mimics the native environment of the membrane proteins [67].

A third method to create a membrane-like environment for housing membrane proteins is by inserting them into nanodiscs [68]. Nanodiscs are macromolecular structures that spontaneously assemble when lipids are mixed with apolipoprotein A1 or B [69,70] (Figure 2C). Approximately 1000 phospholipids molecules associate as a lipid bilayer and two copies of apolipoprotein wrap around the bilayer, thereby protecting the hydrophobic chains of the phospholipid molecules. Membrane proteins can be inserted *de novo* into the nanodiscs through coupled *in vitro* transcription-translation [71]. Alternatively, detergent-soluble membrane proteins can be inserted into nanodiscs by dialyzing away the detergent. Due to the small size of the nanodiscs (*i.e.*, 10 nm diameter), only one copy of a membrane protein is inserted per nanodisc [72,73,74]. A number of different membrane proteins have been reconstituted into nanodiscs and demonstrated to be functional [75,76,77,78].

While at this point in time, there are only three reports of using nanodiscs to affinity select recombinant antibodies to "native" membrane proteins [79,80,81], we anticipate that it will be a fruitful format in future efforts. To aid in the affinity selection process, a biotin tag can be enzymatically or chemically attached to the apolipoprotein A1 or B protein [82], before or after assembly of nanodiscs. This allows for the indirect capture of the nanodiscs to streptavidin-coated magnetic beads for affinity selection, while not altering epitopes in the target membrane protein. Affinity reagents that recognize the apolipoprotein or lipids in the nanodisc can be removed from the library by negative selection, where the phage library is incubated with nanodiscs that do not contain the target protein. This approach should yield affinity reagents that recognize the extracellular and intracellular domains of functional transmembrane proteins.

### 2.3. Virus-Like Particles

**Figure 3:**
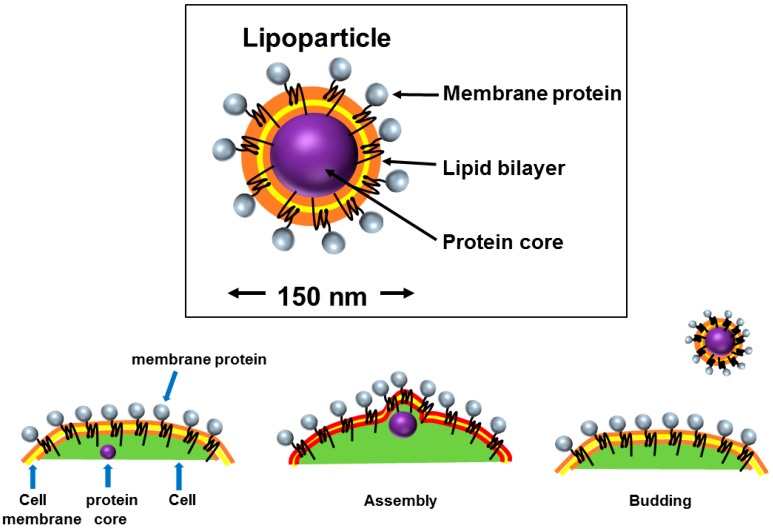
The formation of a virus-like particle (VLP). Mammalian cells expressing the capsid of a retrovirus are transiently transfected with an expression vector for the membrane protein of interest. The retroviral capsid protein directs budding from the plasma membrane of the cell, taking membrane proteins with it, including the overexpressed membrane protein of interest.

**Figure 4:**
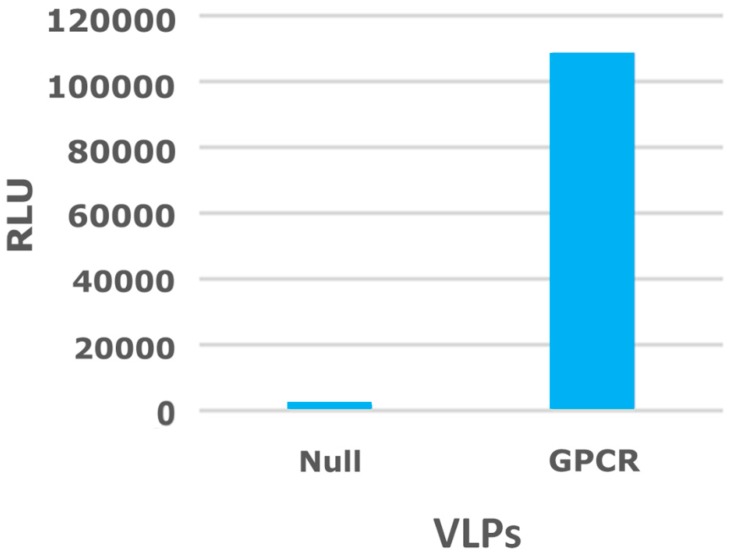
Binding of an antibody to virus-like particles (VLPs) that express a G-protein coupled receptor (GPCR). A mouse monoclonal antibody was tested for binding to biotinylated null VLPs or biotinylated VLPs expressing a GPCR. VLPs were incubated with streptavidin-coated magnetic beads and blocked with milk. The monoclonal antibody was incubated with each VLPs attached to beads, washed with PBS + 0.01% tween (PBS-T), and then incubated with anti-mouse-Horseradish peroxidase (HRP). After final washes with PBS-T, the beads were transferred to an enzyme-linked immunosorbent assay (ELISA) plate and incubated with a chemiluminescent reagent, followed by reading of ELISA signal in a plate reader. RLU is the abbreviation for relative luminescence unit.

A virus-like particle (VLP) (Figure 2D) is similar to a budded enveloped virion that contains only the viral capsid protein that directs and executes viral budding from the cell membrane and is devoid of the viral genome and all other viral proteins (Figure 3). VLPs can be biotinylated either on the membrane lipid or on a membrane protein [83,84] and can be purchased commercially. VLPs are sturdier than intact mammalian cells, thus offering advantages during the affinity selection process by allowing for the addition of detergents, such as Triton X-100 [85,86,87] or Tween 20 [87,88], to the wash buffer to decrease non-specific binding. To test the expression of a GPCR protein on VLPs surface, an enzyme linked immunosorbent assay (ELISA) was performed with a monoclonal antibody, in which the antibody bound to GPCR-expressed VLPs but not to the null (*i.e.*, not expressing the target receptor) VLPs (Figure 4).

Moreover, since VLPs can be biotinylated, competition with unmodified null VLPs can be performed during the affinity selection process to minimize recovery of virions that display antibodies binding to the VLPs themselves. There are multiple advantages associated with VLPs for phage display. VLPs are much more stable than micelles or liposomes [89,90] and can be stored for a long period of time without losing their functions. The multivalent display [91] of the membrane proteins on VLPs may help retain the binding clones through avidity during the affinity selection experiments, which may facilitate the discovery of even weak antibody binders. One major disadvantage of using VLPs is their cost. Commercially available VLPs are expensive, and, although they can be made in-house, it can be laborious to optimize each receptor target for high expression and to quality control for biotinylation levels.

### 2.4. Native Cells

Cultured cells, which endogenously express the target proteins, are often used in affinity selection experiments with phage-display libraries. The cells are kept intact, and virions displaying antibody fragments will only be retained, if they bind to an antigen on the surface of the cells. For example, monolayers of cells are incubated with a phage-display library, non-binders are washed away, and bound virions are recovered by acid elution (*i.e.*, pH 2) or with a detergent that lyses the cells. After repeated rounds of affinity selection, the output is examined: binding of individual clones to cultured cells can be confirmed by an ELISA [92] or through phage titering [93,94]. Generally, one looks for a signal several fold higher for a particular cell line, compared to another cell line that does not express the membrane protein of interest. The main advantages of using native cells for generating antibodies against membrane proteins are that the target is in its native, physiologically-relevant state and is properly post-translationally modified. One disadvantage is due to the presence of many non-relevant targets on the cell surface, the affinity selection process is inefficient with high background of non-specific binders.

### 2.5. Engineered Cell Lines

In addition to working with native cells, one can transfect cells with plasmids that overexpress target membrane proteins on the cell surface. The major advantage of this approach is that matched cell lines can be generated—one expressing the cell surface protein of interest and the other one lacking the protein—which permits negative selection of phage-display antibodies that recognize non-relevant cell surface antigens.

**Figure 5:**
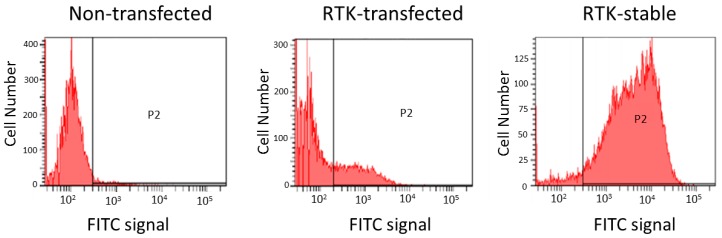
Cell surface expression in transiently-transfected or stably transfected cells. Human embryonic kidney 293 (HEK-293) cells were transiently transfected with an expression vector for a receptor tyrosine kinase (RTK) using a cationic lipid transfection reagent. Twenty-four hours post-transfection, the cells were stained with an anti-RTK monoclonal antibody and fluorescein isothiocyanate (FITC)-labeled anti-mouse antibody and analyzed by fluorescence-activated cell sorting (FACS). The FITC signal for the transfected cells was compared to non-transfected cells or cells stably transfected to express the target RTK. The percentage of FITC-positive cells was 94% for the stable cell line *versus* 27% for the transfected cells.

**Figure 6:**
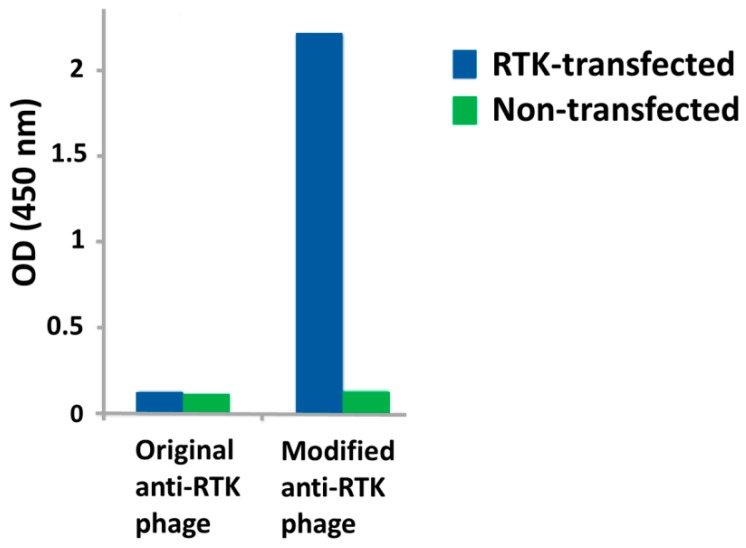
Cell enzyme-linked immunosorbent assay (ELISA) sensitivity using monovalent *versus* multivalent phage display system against transiently transfected cells. Human embryonic kidney 293 (HEK-293) cells were transiently transfected with an expression construct for a receptor tyrosine kinase (RTK). Phage displaying a scFv specific for the RTK was produced using either a standard helper phage (M13K07) or a modified helper phage (hyperphage) for monovalent or multivalent display of the scFv on the M13 virion, respectively. The phage produced using the monovalent display system showed no binding to the transfected cells in a cell ELISA. In contrast, the phage produced using the multivalent display system showed a dramatic increase in binding to the RTK-expressing cells.

In this approach, one can use either transiently transfected cells or stable cell lines (Figure 5). There are advantages and disadvantages with both types of cells. While target protein expression typically occurs within 24–48 h of transfection [95], the expression level is variable, with high levels of protein expression sometimes leading to cell death, thus decreasing the population of cells expressing the protein of interest over time [96]. The efficiency of transfection is also variable and is cell-type dependent. There are many approaches for transfection of mammalian cells, including cationic lipids, electroporation, *etc.* and each method will require optimization for the specific cell type [96,97]. Despite these disadvantages, transiently transfected cells can be used for affinity selection of phage-display antibodies that bind to cell surface receptors, especially when coupled with a multivalent phage display system, which increases the sensitivity of the phage for its target (Figure 6) through avidity [98].

For generating stable cells, it can take weeks to months to produce a cell line that expresses a satisfactory level of a membrane protein, but once identified, a single cell can be clonally expanded to express uniformly high levels of protein at sub-lethal levels with inducible promoter systems [99,100,101,102]. Stable cells can be stored and kept for indefinite amounts of time and may be a source of reliable antigen that allows for repeated experimentation [103]. Certain cell lines, such as Human embryonic kidney 293 (HEK-293) [104,105] and insect cells [34,106], have been used widely for making both transient and stable cells for overexpressing membrane proteins.

## 3. Challenges and Novel Methods for Affinity Selecting Recombinant Antibodies against Membrane Proteins

As the membrane protein of interest represents only a small fraction of available epitopes (*i.e.*, extracellular matrix, phospholipids, glycolipids, *etc.*) on a cell surface, there are several challenges to generate recombinant affinity reagents to membrane proteins. First, when using cell lines in affinity selection experiments, it is difficult to wash cells adequately to eliminate non-specific binding virions; virions can stick to cell surfaces and be trapped by clumped cells. Second, experiments to compete or subtract common epitope between two cell lines are often incomplete. Third, generally the signal-to-noise ratio for binding to cells is often small (*i.e.*, <2). Fourth, even when binders are isolated from whole cell screening, it is formidable to determine what the targets are for the binders.

**Figure 7:**
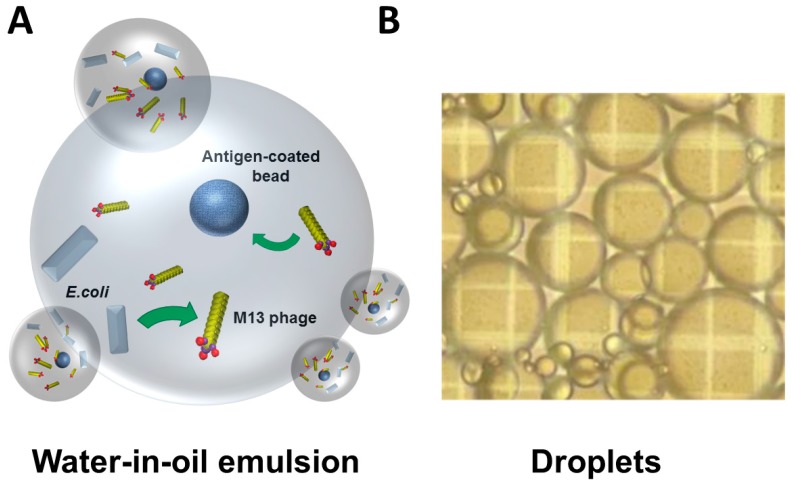
Phage micro-emulsion technology. (**A**) Schematic of phage micro-emulsion technology. A heterodisperse emulsion is produced containing 5–10 phagemid-transduced *E. coli* and 3–6 antigen-coated beads (or target expressing cells) per microdroplet. Phage secreted from M13-transduced *E. coli* that encodes a recombinant antibody (rAb) against the target antigen will attach to the beads (or cells) within the droplet. After overnight incubation, the emulsion is broken, and the beads (or cells) are washed to remove unbound phage. A fluorescein isothiocyanate (FITC)-labelled anti-M13 secondary antibody is added to detect any bound phage, and the FITC-positive beads (or cells) are sorted by fluorescence-activated cell sorting (FACS). (**B**) An image of microdroplets under a light microscope.

To address the above challenges, several new affinity selection methods have been developed. We have previously described a novel approach to phage display that utilizes water-in-oil emulsions (Figure 7) for the identification and isolation of phage displayed scFvs [107]. Using this method, a large library of antibody-displaying virions is bound to beads in individual oil droplets rather than affinity selection on a large mixed population. This method allows one to query clonal populations of amplified phage against the antigen while eliminating competition from high affinity binders. In the emulsion droplets, a highly localized concentration of virions and antigen allows one to isolate a greater diversity of binders during the affinity selection process. This may be critical in the context of a complex antigen source, such as a whole cell or tissue sample, where antibodies against the highly expressed or immunodominant epitopes will outcompete the less abundant or less antigenic ones when bulk affinity selection is performed.

One new method in affinity selection of antibodies for efficiently partition binders over non-specific binding background is called “pathfinding”. This is a two-step method, in which cells are first incubated with a ligand or an antibody that interacts with protein of interest. Such a ligand or antibody is also conjugated to horseradish peroxidase (HRP), and after washing, the cells are incubated with the phage-display library. After eliminating non-specific binding virions with thorough washing, the cells are incubated with biotin-tyramide [108]. All free lysine residues near the HRP-labeled ligand or antibody, including the lysine residues on the virions that display the binding antibody fragments, are biotinylated *in vitro*. Such biotinylated virions can be partitioned away from non-specific binding virions with streptavidin-coated magnetic beads. This method has been successfully used to generate antibodies against C-C chemokine receptor type 5 (CCR5) [109]. One drawback of this method is that the ligand or the existing antibody may interfere with the binding of phage displayed antibodies to the target. As a result, instead of isolating antibodies to the target protein, antibodies binding to the cell surface antigens in close vicinity of the target protein are isolated [110].

To improve affinity selection process with cells grown in suspension, a method termed “Biopanning and Rapid Analysis of Selective Interactive Ligands” (BRASIL) has been developed [111]. This approach involves incubating a suspension of cultured cells with a phage library, followed by centrifuging the cells through an organic non-miscible lower phase, which washes cells free of all virions except those binding to a cell surface target. This method has been used to generate recombinant affinity reagents to an osteosarcoma cell surface antigen [112], Toll-like receptor 2 [113] and a thyroid tumor cell surface marker [114].

To remove the non-specific binders even more efficiently, microfluidic devices have been used for affinity selection [115,116]. Such microfluidic devices utilize continuous flow for washing, which prevents rebinding of non-specific binders. With one of such devices, a phage peptide library was affinity selected against neuropilin-1 on the surface of the human prostate carcinoma cells and several known and novel peptide sequences against neuropilin-1 were isolated [116]. In a side-by-side comparison, the microfluidic device removed non-specific binders and enriched binders much more efficiently than affinity selection with the regular cell suspension.

For multi-pass membrane proteins, here we propose an alternative approach for affinity selection. First, multiple peptide antigens, corresponding to the extracellular loops, N-termini and C-termini of the target proteins, can be designed with the aid of a variety of software tools for predicting protein structures [117,118]. One can use the peptides for affinity selection of a phage-displayed antibody library, and the enriched pool of clones can be mutated by chain shuffling and error-prone PCR [9]. The resulting mutant library can be used to affinity-select cells expressing the endogenous membrane protein of interest. There are several advantages to this approach. First, the initial rounds of affinity selection with peptides will help remove majority of the non-specific binding virions. Second, the copy number of a particular binder enriched by initial affinity selection is much higher than that in the non-selected naïve library, and the extra copies will increase the likelihood of this binder to bind its endogenous antigen on the cell surface and will help it retain during the extensive washing.

## 4. Various Applications of Recombinant Antibodies

Recombinant affinity reagents generated by phage display to membrane proteins have been widely used for basic science research, diagnostics, and therapeutics. One of their most common applications in basic science is for promoting crystallization of membrane proteins to solve three-dimensional structures. Membrane proteins are notoriously difficult to crystallize due to their heterogeneous nature. In addition, the detergent micelle or liposome used for solubilizing and stabilizing the membrane proteins can interfere with protein crystallization [119]. Recombinant affinity reagents not only stabilize the membrane proteins to increase conformational homogeneity but also create extra crystal contact surface, both of which promote crystallization [119]. With such an approach, Fabs have been generated by phage-display to assist the crystallization of K+ channel [120,121]. Nanobodies have been generated for crystalizing two membrane proteins, including the β2 adrenergic receptors at its active conformation [122] and the ATP-binding cassette (ABC) transporter P-glycoprotein [123]. This same approach has been applied to phage-displayed fibronectin type III (FN3) monobodies for solving structures of fluoride ion channel [64], and applied to designed ankyrin repeat proteins (DARPins) to crystallize multidrug exporter AcrB [124].

Efforts have also been undertaken to generate recombinant antibodies for diagnostic purposes. Recombinant antibodies have been generated for diagnosing infectious diseases, such as influenza [125,126,127] and encephalitis [128]. Antibodies [129,130,131] and nanobodies [132,133] have also been generated for cancer diagnosis. To aid cardiovascular imaging, a high affinity nanobody has been engineered to recognize vascular cell adhesion molecule-1 (VCAM1) [134].

One of the most important applications of recombinant affinity reagents is for treating diseases as therapeutics. As of 2016, there are seven phage-display derived drugs that have been approved by Food and Drug Administration (FDA) in the USA. The approved list includes the global best-selling drug, Adalimumab (also known as Humira), a recombinant antibody used to treating autoimmune diseases [135]. Two of the approved protein drugs targeting membrane proteins are Herceptin [136], which recognizes human epidermal growth factor receptor 2 (HER2) for treating breast cancer, and Romiplostim, which binds to thrombopoietin for treating Idiopathic thrombocytopenic purpura [137]. Besides the seven approved drugs, there are dozens of phage-display derived reagents in different phases of clinical trial [138]. In summary, phage display becomes one of the most popular display technologies in engineering proteins for therapeutic purpose.

In this review, we summarize the some of the latest progress of engineering antibody or its mimics against membrane proteins for applications in basic research, diagnostics and therapeutics. We give details of several formats for presenting the membrane proteins for affinity selection and several new methods to improve the efficiency of the affinity selection process. We expect that with all these progress, applications of recombinant antibodies generated by phage display will continue to expand and they will make significant impact on various fields of life science research.
